# Efficacy of steroid addition to multimodal cocktail periarticular injection in total knee arthroplasty: a meta-analysis

**DOI:** 10.1186/s13018-015-0214-8

**Published:** 2015-05-22

**Authors:** Xinyu Zhao, Jun Qin, Yang Tan, Rahul Mohanan, Dongcai Hu, Liaobin Chen

**Affiliations:** Department of Orthopaedics, Zhongnan Hospital of Wuhan University, School of Medicine, Wuhan University, 169 Donghu Road, Wuhan, Hubei Province 430071 China

**Keywords:** Total knee arthroplasty, Steroid, Periarticular injection, Meta-analysis

## Abstract

**Background:**

Total knee arthroplasty (TKA) has been reported to be the most successful treatment for patients with advanced osteoarthritis, however, early postoperative pain has become an unresolved issue. The aim of this Meta-analysis is to evaluate the efficacy and safety of steroid addition to multimodal cocktail periarticular injection (MCPI) in patients undergoing TKA.

**Method:**

Clinical randomized controlled trials concerning the efficacy and safety of MCPI containing steroids in TKA published up to December 2014 were retrieved from PubMed, Cochrane library, EMbase databases. The methodological quality of the included studies was assessed by the 12-item scale. Data analysis was performed using StataSE12.0.

**Results:**

Six randomized controlled trials involving a total of 567 patients were assessed; the steroid group included 305 patients, and the control group included 262 patients. The meta-analysis showed that MCPI with steroids in TKA significantly reduced postoperative pain; duration of time required to perform straight-leg raising and length of hospital stay was (*P* < 0.05). Neither the early postoperative nor the long-term range of motion of knee showed any statistical difference between the non-steroid and steroid group (*P* >0.05). For safety, steroids did not increase the incidence of postoperative infection and wound oozing (*P* >0.05); no tendon rupture was reported up to now. In addition, steroids did not decrease the postoperative drainage through the reduction of prostaglandins (*P* >0.05).

**Conclusion:**

For patients undergoing TKA, the addition of steroids to MCPI improved the analgesic effect and was proved to be highly safe. The duration of time required to perform straight-leg raising and length of hospital stay was significantly reduced. However, MCPI with steroids neither increased the early postoperative range of motion (ROM) or the long-term ROM of knee, nor did it reduce the postoperative drainage. However, the best results are acquired in patients without any altered immunological status.

## Background

Total knee arthroplasty (TKA) has been reported to be the most successful treatment for patients with advanced osteoarthritis; however, the early postoperative pain has become an unresolved issue [1]. Statistically, postoperative pain following TKA is severe in approximately 60 % of patients and moderate in approximately 30 %. Many patients gave up TKA as they were concerned about the postoperative pain [2]. Good postoperative analgesia improves patient satisfaction, facilitates earlier rehabilitation, reduces length of hospital stay, and decreases potential for postoperative complications like deep vein thrombosis or pneumonia [3]. Although good pain relief could be achieved by continuous epidural anesthesia, femoral nerve block, and patient controlled analgesia, the side effects are quite worrisome. It includes muscle weakness, haematoma, respiratory depression, urinary retention, nausea, and vomiting which are all unfavorable for early rehabilitation and increases chances for venous thromboembolism [4, 5]. Results from recent studies indicated that multimodal cocktail periarticular injection (MCPI) during TKA had shown good postoperatively pain relief, improvement in range of motion (ROM) and reduction of postoperative complications [6]. However, there is no gold-standard protocol for the drug composition and quantity of the cocktail injection. Recent studies have suggested that the addition of steroids to MCPI might decrease the local inflammatory response following surgical trauma. Thereby it resulted in decreased edema and blood loss owing to the reduced production of prostaglandins which causes vasodilation. Moreover, the patients may also enjoy a permanently better ROM as steroids reduce the postoperative fibrosis and scarring [7–9]. However, many surgeons refrain from using periarticular steroids as there is a possible complication of postoperative infection and patellar tendon rupture [10].

Recently, several randomized controlled trials (RCTs) have compared the efficacy of MCPI with or without steroids in TKA, but the conclusions were controversial. Ikeuchi et al*.* [11] reported that MCPI with steroids resulted in significant early pain relief and rapid recovery in TKA. Kown et al. [12] observed that it just had an additional pain-relieving effect on the night of the operation. However, Christensen et al. [10] stated that MCPI with steroids can only reduce the length of the hospital stay following TKA, but it appears to improve neither pain relief nor ROM in the early postoperative period. Until now, no meta-analysis in this field has focused on it. Thus we undertook a meta-analysis to evaluate the efficacy and safety of the addition of steroids to MCPI during TKA in order to provide a reference for surgeons.

## Methods

### Search strategy

The PubMed, Embase, and the Cochrane Central Register of Controlled Trials were searched from their earliest entries up to December, 2014. The search strategy was (((((((((random*[Title/Abstract]) OR “Randomized Controlled Trial” [Publication Type])) AND (((“Arthroplasty, Replacement, Knee”[Mesh]) OR ((((knee arthroplasty[Title/Abstract]) OR knee replacement[Title/Abstract]) OR knee replacements[Title/Abstract]) OR knee prosthesis[Title/Abstract])))))))))) AND (((((((“Steroids”[Mesh]) OR ((steroid[Title/Abstract]) OR corticosteroid[Title/Abstract])))))) OR ((“Glucocorticoids”[Mesh]) OR glucocorticoid [Title/Abstract])). The reference list of the relevant literatures were also reviewed manually for any further relevant studies. Languages were not restricted in this search.

### Inclusion criteria and exclusion criteria

Inclusion criteria were as follows: (1) The target population consisted of patients undergoing unilateral primary TKA; (2) The interventional group should merely have a steroid in addition to the control group; (3) The outcomes were analyzed with respect to visual analogue scale (VAS), ROM of knee, postoperative drainage, duration of time required to perform a straight-leg raise (SLR), length of hospital stay, incidence of complications such as postoperative infection, and wound oozing; (4) The methodological criterion was prospective RCT.

Exclusion criteria were as follows: (1) Patients undergoing bilateral TKA, unicondylar knee arthroplasty or revision; (2) Other difference between the steroid group and control group besides the administration of steroids; (3) Animal studies.

### Date extraction and assessment of methodological quality

After the consecutive procedures of screening of titles and abstracts, obtaining the full text of each article and reviewing them, articles that met the eligibility criteria and did not meet the exclusion criterias were selected to be included. Data were extracted and collated independently by two authors (ZXY and QJ), including author, published year, sample size, patient age, sex, body mass index, drug composition and quantity of the cocktail, injection method, other intervention protocol, and the aforementioned outcome indexes. The data of a published updated study involving the same cohort of patients was extracted synthetically. The original investigators were contacted when requisite data were lacking in the publications. The methodological quality of each included RCT was assessed by two observers independently by the 12-item scale [13]; trials with a score of seven or more were considered high quality, more than four but less than seven was considered moderate quality, and less than four was considered low quality. Disagreements were evaluated by the means of kappa text and were resolved by discussing with the corresponding author (CLB).

### Statistical methods

The meta-analysis was conducted with StataSE12.0 software. The weighted mean difference (WMD) and 95 % confidence interval (CI) were calculated for continuous data, and the relative risk (RR) and 95 %CI were calculated for dichotomous data. The statistical heterogeneity was tested with the chi-square test and *I*2. If heterogeneity was low (*P* > 0.1, *I*2 < 50 %), a fixed-effects model was used. If heterogeneity was significant (*P <* 0.1, *I*2 > 50 %), sensitivity analysis and subgroup analyses were conducted to find the source of the heterogeneity. If the heterogeneity could not be eliminated, a random-effects model would be used when the result of meta-analysis had clinical homogeneity, or a descriptive analysis would be used.

### Source of funding

No external funding was received in support of this study.

## Results

### Study characteristics

**Fig. 1:**
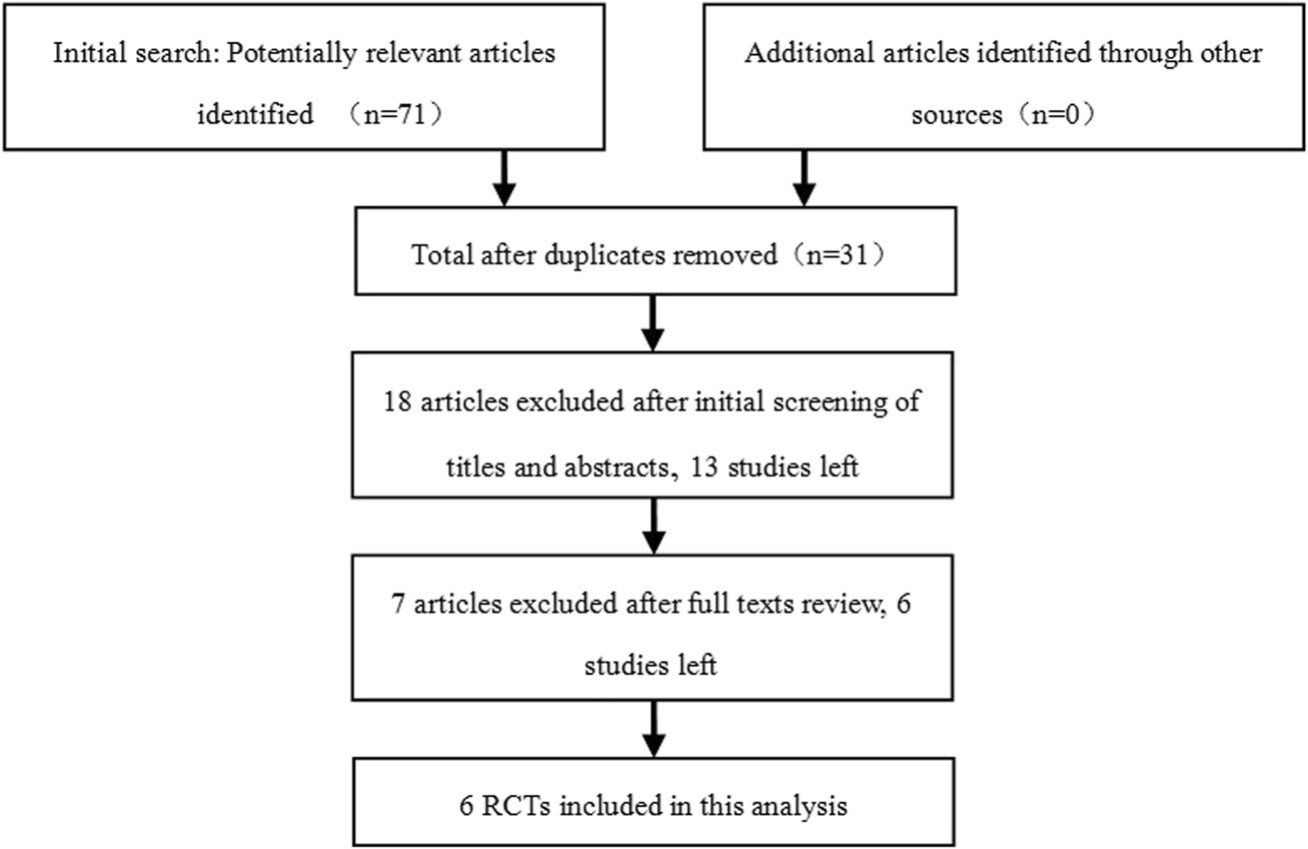
Flow chart summarizing the selection process of randomized control trials

A total of 71 potential articles were identified and screened for the meta-analysis. After screening of titles and abstracts, obtaining the full text of each article and reviewing them, six RCTs were selected for this meta-analysis [10–12, 14–16] (Fig. 1). The cumulative sample size of 567 unilateral primary TKA comprised of 305 with steroids and 262 without steroids. All cases were successfully followed up except one patient in the steroid group who died 25 days postoperatively as a result of complications associated with deep knee-joint sepsis. The main characteristics of the included studies were summarized in Table 1, and the literature-exclusion procedure was depicted in Fig. 1. The methodological quality of the included RCTs was assessed with the 12-item scale (Table 2); the results showed that the average score for the quality of included studies was 11.67 ± 0.52, and all RCTs were of high quality.

**Table 1:** Details on the included studies in the meta-analysis

Author	Year of publication	Sample size	Patient mean age	Sex (M/F)	BMI	Steroid/control group	Drain/blocks/anesthesia	Cocktail	Steroid Used	Administration Method	Type of Knee arthroplasty	Other intervention protocol
Kwon	2014	76	69.3	0/76	25.9	76/76	?/none/SA	10 mg morphine, 300 mg ropivacaine,30 mg ketorolac, 300 μg of 1:1000 epinephrine	40 mg Triamcinolone	SLIA IAPC	Cemented TKA	Preoperative celecoxib, ultracet,pregabalin
											Unresurfaced patella	Postoperative PCA
Yue	2013	72	69.75	8/64	25.685	36/36	All drains/none/GA	0.75 % ropivacaine 30 ml, 1:1000 adrenaline 0.5 ml	7 mg Betamethasone	SLIA IAPC	TKA(unclassified)	
												Postoperative PCA, celecoxib
Ikeuchi	2014	40	76.5	6/34	?	20/20	All drains/none/GA	20 ml of 0.75 % ropivacaine, 400 mg of isepamicin	6.6 mg Dexamethasone	Peri-articular tissues	Cemented TKA	
											Unresurfaced patella	Postoperative PCA, loxoprofen
Chia	2013	127	66.93	?	31.15	84/43	None/none/SA	0.2 % ropivacaine and 100 ml 1:1000 adrenaline	Triamcinolone	SLIA IAPC	Cemented TKA,	
									40 mg in subgroup1, 80 mg in subgroup2		Resurfaced patella	Postoperative celecoxib
Seah	2011	100	66.65	?	27	50/50	?/none/SA or GA	0.5 ml/kg of 1:200,000 epinephrine and 0.5 % bupivacaine	40 mg of Triamcinolone	SLIA IAPC	Cemented TKA,	
												Postoperative PCA
Christensen	2009	76	65.508	23/53	33.9	39/37	?/FNB/GA	80 mg of bupivacaine, 4 mg morphine, 300 mg epinephrine, 100 mg clonidine, 750 mg cefuroxime	40 mg Methylprednisolone	SLIA IAPC	TKA(unclassified)	Preoperative elecoxib, oxycodone or acetaminophen

*SA* Spinal Anesthesia, *GA* General Anesthesia, *SLIA IAPC*, Systematic Infiltration Including Anterior and Posterior Capsule, *TKA* Total knee Arthroplasty, *PCA* Patient Controlled Analgesia, *FNB*, Femoral Nerve Block, ? Unknown

**Table 2:** 12-item scale critical appraisal scores

Author	12-item scale critical appraisal score												
	1	2	3	4	5	6	7	8	9	10	11	12	Total
Kwon 2014 [12]	Y	Y	Y	Y	Y	Y	Y	Y	Y	Y	Y	Y	12
Yue 2013 [14]	Y	Y	Y	Y	Y	Y	Y	Y	Y	Y	Y	Y	12
Ikeuchi 2014 [11]	Y	Y	Y	Y	Y	Y	Y	Y	Y	Y	Y	Y	12
Chia 2013 [16]	Y	Y	Y	Y	Y	Y	Y	Y	Y	Y	Y	Y	12
Seah 2011 [15]	Y	Y	Y	N	Y	Y	Y	Y	Y	Y	Y	Y	11
Christensen 2009 [10]	Y	Y	Y	Y	N	Y	Y	Y	Y	Y	Y	Y	11

12-item scale criteria: (1) Method of randomization; (2) Concealed allocation; (3) Patient blinding; (4) Provider blinding; (5) Outcome assessor blinding; (6) Drop-out rate; (7) Patient allocated as plan; (8) Free of selective outcome reporting; (9) Same baseline; (10) Co-interventions avoided or similar; (11) Acceptable compliance; (12) Same time of outcome assessment. Y = Yes, N = No, A trial with a score of seven or more was considered high quality, more than four but no more than seven was considered moderate quality, and no more than four was considered low quality

### Postoperative VAS

Pain score was assessed by VAS on the operative night, and at postoperative days 1, 2, 3, and 7 in 5 [10–12, 14, 15] of the 6 studies. The results of the meta-analysis on the operative night and postoperative day one appeared heterogeneous (*P <* 0.1, *I*2 > 50 %); sensitivity analysis did not detect the source of heterogeneity, and subgroup analyses divided by possible diversity failed to eliminate the heterogeneity. Then we found that, regardless of the exclusion or inclusion of every study, the results were all the same and had clinical agreement, so we conducted the meta-analysis by the random-effects model for the reason that all studies were of high quality. The fixed-effects model was used in the rest postoperative period as heterogeneity was not detected (*P* > 0.1, *I*2 < 50 %). The pooling results indicated that the VAS at postoperative days 2, 3, and 7 of the steroid group were significantly lower than those of the control group (*P* < 0.05, Table 3); however, no statistical difference of VAS on the operation night and at postoperative day one was detected between the two groups (*P* > 0.05, Table 3).

**Table 3:** Comparison of postoperative pain VAS scores between the steroid and control group

Postoperative period	Weighted mean of VAS in the steroid group	Weighted mean of VAS in the control group	WMD	[95 % CI]		*P* of heterogeneity	*I*2	Selected model	Overall *P*
Night of operation	2.270	2.888	−0.328	−0.914	0.258	0.006	80.3 %	Random-effects model	0.272
Day 1	3.362	3.805	−0.630	−1.321	0.061	0.001	87.1 %	Random-effects model	0.074
Day 2	2.354	2.865	−0.300	−0.504	−0.096	0.249	28.0 %	Fixed-effects model	0.004
Day 3	2.394	2.882	−0.568	−0.868	−0.268	0.194	36.3 %	Fixed-effects model	0.001
Day 7	2.264	2.693	−0.310	−0.496	−0.125	0.520	0 %	Fixed-effects model	<0.001

### Postoperative ROM

**Fig. 2:**
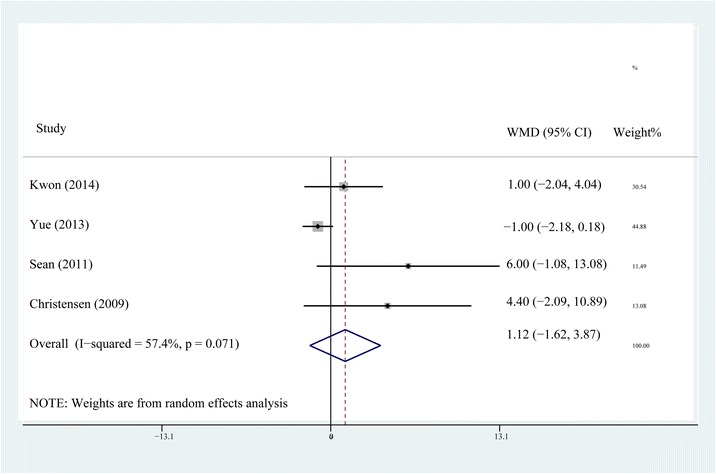
Comparison of knee ROM between the steroid and control group at the first postoperative day

**Fig. 3:**
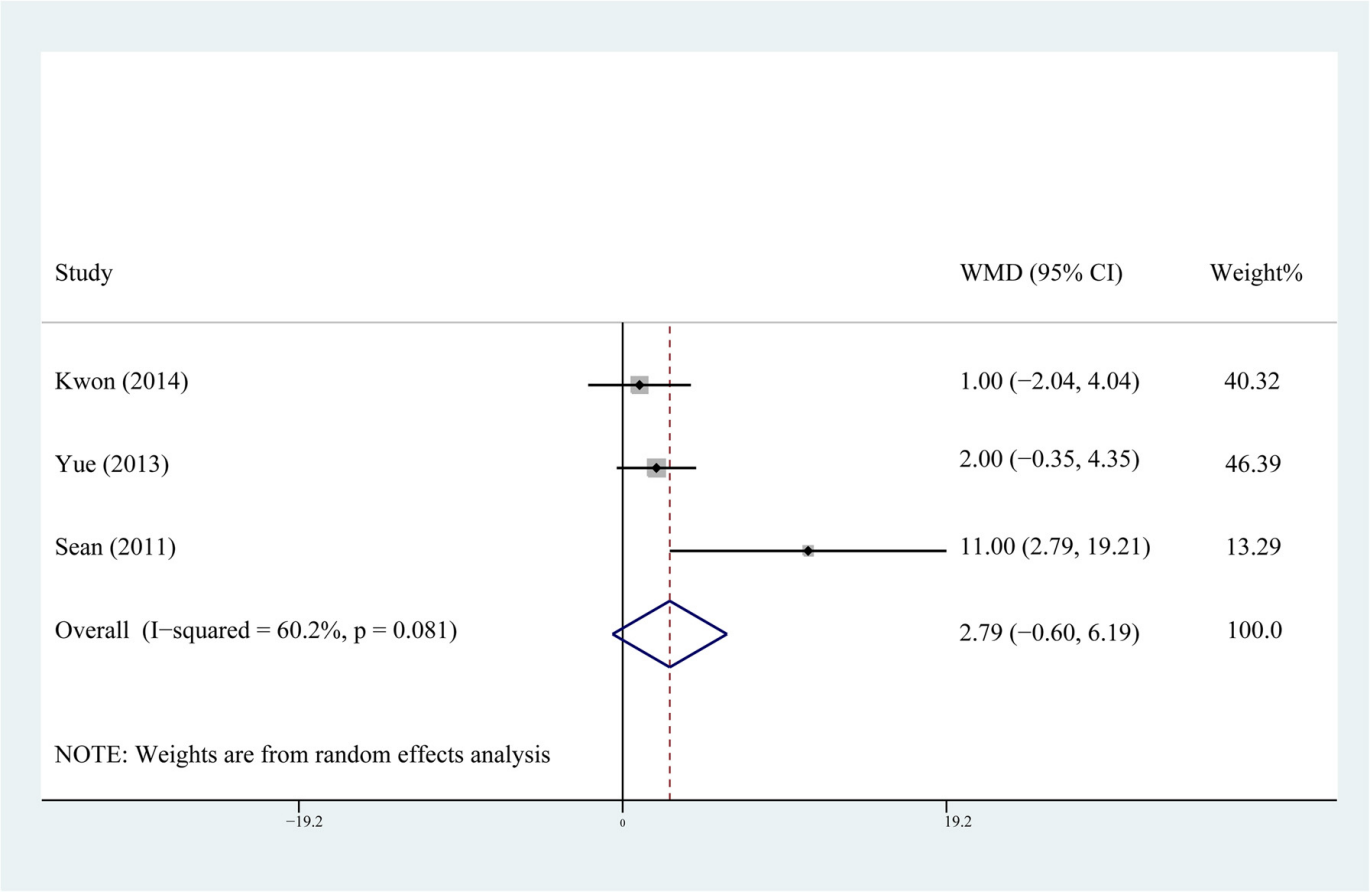
Comparison of knee ROM between the steroid and control group on the second postoperative day

**Fig. 4:**
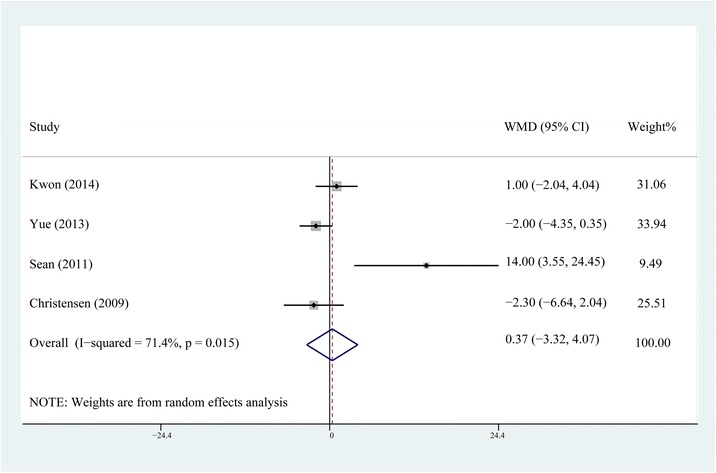
Comparison of knee ROM between the steroid and control group on the third postoperative day

**Fig. 5:**
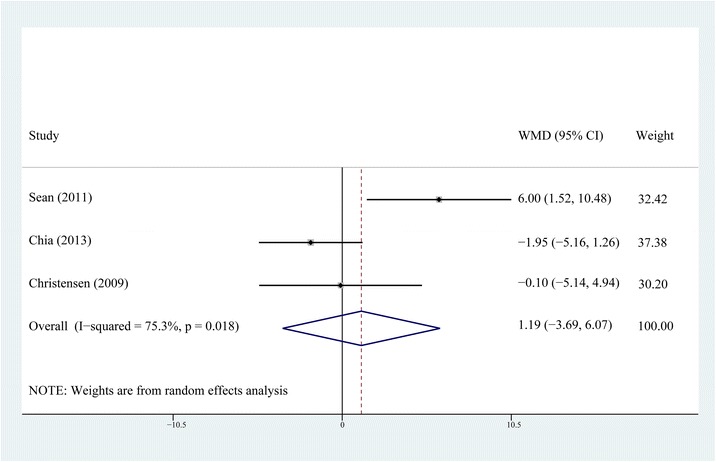
Comparison of knee ROM between the steroid and control group at 3 months postoperatively

ROM at postoperative days 1, 2, 3, and month 3 was reported in four of the six studies [10, 12, 14, 15]. Heterogeneity was detected in every postoperative period. Sensitivity analysis and subgroup analysis failed to eliminate the heterogeneity. The random-effects model was selected for the reason that no matter what the exclusion or inclusion of every study, the results were all the same and had clinical agreement. The results of meta-analysis showed that the ROM of knee at postoperative days 1, 2, 3, and month 3 did not show any significant difference between the steroid group and the control group (*P* > 0.05, Figs. 2, 3, 4, and 5).

### Postoperative drainage

**Fig. 6:**
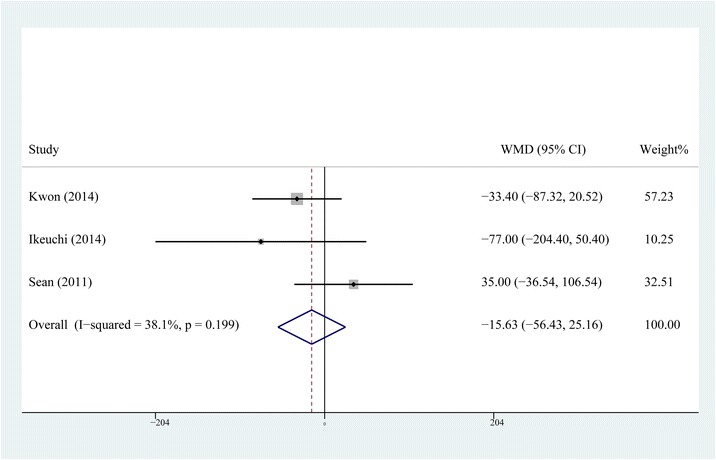
Comparison of postoperative drainage between the steroid and control group

Three papers [11, 12, 15] including 146 patients in the steroid group and 146 patients in the control group described the postoperative drainage; a fixed-effects model was used as no heterogeneity was detected (*P* = 0.199, *I*2 = 38.1 %). The pooling result showed no statistical difference of postoperative drainage between the two groups (WMD = −15.63, 95 % CI, −56.43 ~ 25.16, *P* = 0.453, Fig. 6).

### Period of time required to perform a straight-leg raise

**Fig. 7:**
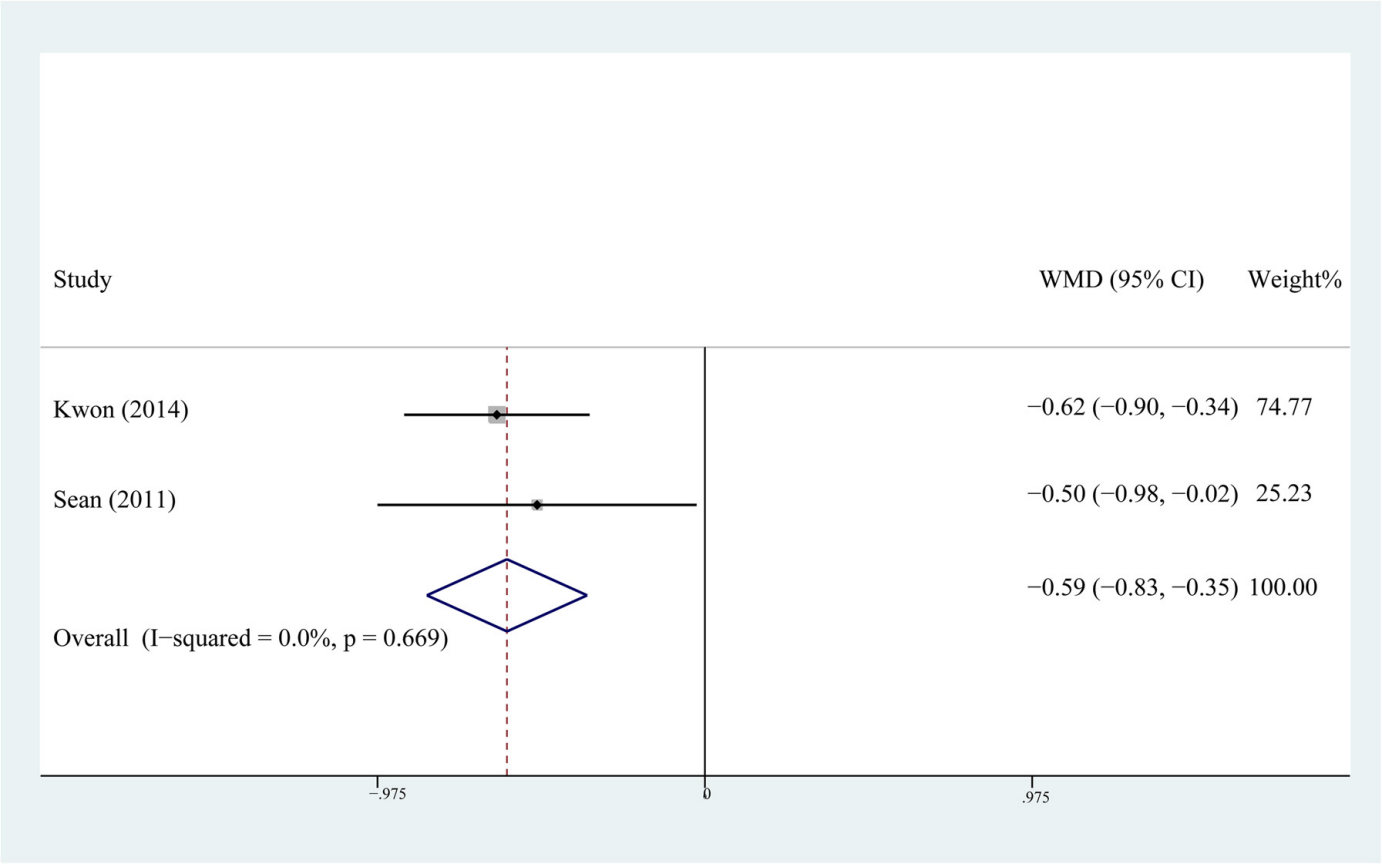
Comparison of the period of time required to perform a straight-leg raise between the steroid and control group

The period of time required to perform a straight-leg raise was reported in two [12, 15] of the six papers, including 126 patients in the steroid group and 126 patient in the control group. The fixed-effects model was selected as no significant heterogeneity was found (*P* = 0.669, *I*2 = 0 %). The result of meta-analysis indicated that patients in the steroid group could perform a straight-leg raise significantly earlier than those in the control group (WMD = −0.590, 95 % CI, −0.829 ~ −0.351, *P* < 0.001, Fig. 7).

### Length of hospital stay

**Fig. 8:**
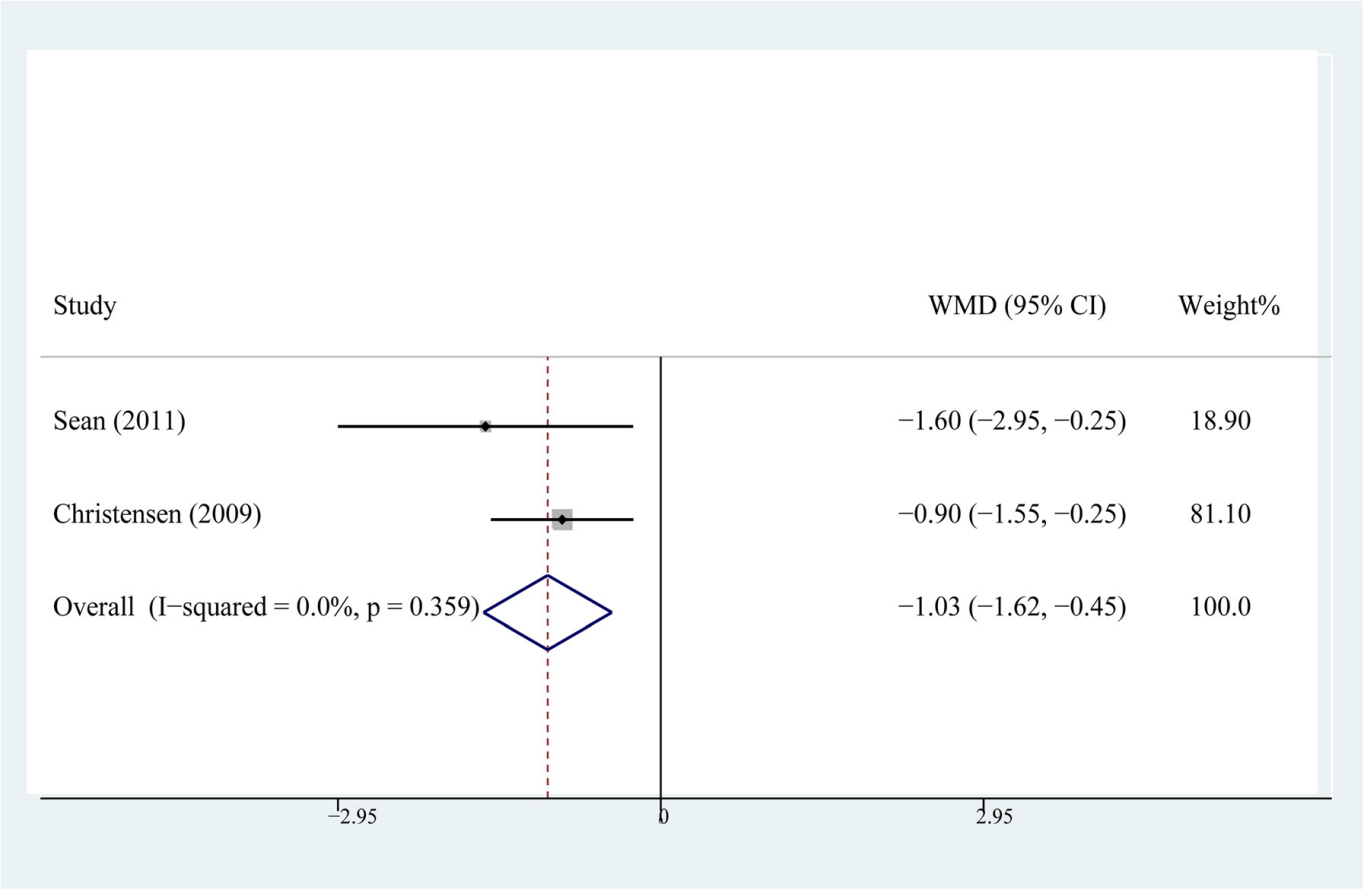
Comparison of the length of hospital stay between the steroid and control group

The length of hospital stay was recorded in two studies [10, 15] including 89 patients in the steroid group and 87 patients in the control group. The fixed-effects model was used as no heterogeneity was detected (*P* = 0.359, *I*^2^ = 0 %). The results of meta-analysis indicated that the length of hospital stay of the steroid group was significantly shorter than that of the control group (WMD = −1.032, 95 % CI, −1.618 ~ −0.447, *P* < 0.001, Fig. 8).

### Incidence of complications

**Fig. 9:**
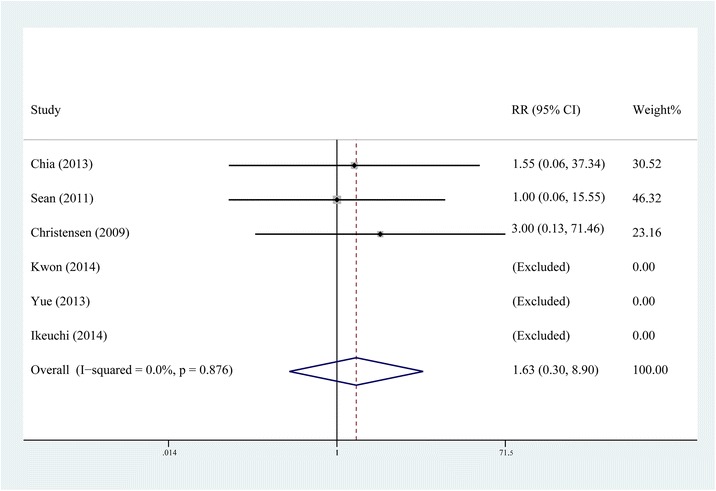
Comparison of the rate of postoperative infection between the steroid and control group

**Fig. 10:**
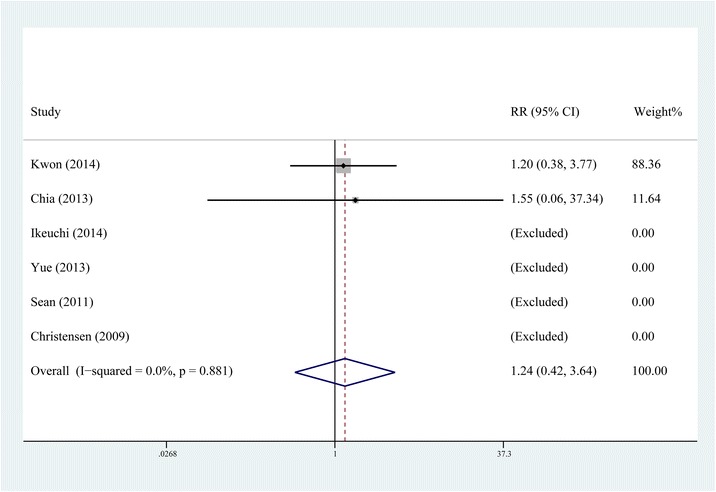
Comparison of the rate of postoperative wound oozing between the steroid and control group

All six studies [10–12, 14–16] reported the incidence of complications such as postoperative infection and wound oozing. The fixed-effects model was selected, respectively, as no significant heterogeneity was found in the results of these two indexes (*P* > 0.1, *I*2 < 50 %). The pooling results manifested that the incidence of postoperative infection had no statistical difference between the steroid group and control group (RR = 1.632, 95 % CI, 0.299 ~ 8.900, *P* = 0.571, Fig. 9) and neither did the incidence of wound oozing (RR = 1.241, 95 % CI: 0.423 ~ 3.641, *P* = 0.694, Fig. 10). For RCTs in our study, the inclusion criteria for four of all six were patients undergoing primary TKA for osteoarthritis; patients with co morbidities resulting in altered immunological status such as history of uncontrolled diabetes mellitus, immunocompromised renal failure, ipsilateral deep-knee infection, and hypersensitivity to one or more drugs included in the cocktail; major psychological ailments or any other systemic conditions which is even a contraindication for a normal TKA were excluded from all the RCTs.

## Discussion

MCPI has been particularly effective in relieving pain, enabling earlier rehabilitation, and improving postoperative ROM, meanwhile, it reduces the complications of other pain management modalities such as continuous epidural anesthesia, femoral nerve block, and patient controlled analgesia [1, 2, 17, 18]. However, the drug composition and quantity of MCPI still has no gold-standard protocol. Considering the efficacy of steroids in decreasing the local inflammatory response following surgical trauma and reducing the postoperative fibrosis and scarring, several RCTs comparing the effects of MCPI with or without steroids have been published, but these studies pointed to different end results [9–12, 14–16, 19]. Although the existing RCTs have confirmed the safety of steroids, many surgeons still hesitate to use a drug which is thought to increase the risk of catastrophic complications like infection and patellar tendon rupture [20–23].

The results of this meta-analysis indicated that the addition of steroids might not further improve the analgesic efficacy of MCPI in the hyperacute phase, which included the operative night and postoperative day one. However, the application of steroids significantly relieved the postoperative pain after that phase, reducing the time required to perform a straight-leg raise and length of hospital stay. As the included RCTs in our study only evaluated the efficacy of steroids in MCPI, the pooling results of our study proved that steroids could further improve the analgesic effect of MCPI. The suppression of inflammatory reaction by periarticular steroid administration is explained by the cascade inhibiting the cytoactivity of immunocytes by blocking the synthesis of phospholipase A2 and thereby reducing the production of inflammatory mediators and cytokines [7]. Ng et al. [19] and Ikeuchi et al. [11] reported the local and systemic anti-inflammatory effects of steroids, which were supported by evaluating interleukin-6 in drain fluid and serum C-reactive protein. The forest plot of the seventh postoperative day showed a better analgesic effect for the steroid group, which made us believe that MCPI with steroids provided prolonged analgesic effects which could be maintained for at least seven days. It also supported the possibility of steroids itself playing a pivotal role, as the effect of local anesthetics should have had already disappeared by that time.

The forest plots demonstrate that neither the early postoperative ROM nor the long-term ROM of knee showed any difference between the non-steroid and steroid group. For the reason that TKA is a major trauma, the analgesic effect is insufficient for a better ROM during the early postoperative period even if an earlier straight-leg rise and a shorter length of hospital stay are achieved by MCPI with steroids. There is no statistical difference in ROM between steroid and non-steroid group at the third postoperative month, which means MCPI with steroids, might not increase the ROM as it does not decrease postoperative fibrosis and scarring at the respective time frame.

For safety, many surgeons refrain from using periarticular steroids, concerned about the risk of postoperative infection and wound problems [24, 25]. Among the included RCTs in our study, the inclusion criteria for four of all six were patients undergoing primary TKA for osteoarthritis. Almost all the RCTs excluded patients with a past history of uncontrolled diabetes mellitus, immunocompromised renal failure, ipsilateral deep-knee infection, and hypersensitivity to one or more drugs included in the cocktail, major psychological ailments or any other systemic conditions which is even a contraindication for a normal TKA [10–12, 14–16]. The result of our meta-analysis indicated that the steroid addition to MCPI would not increase the incidence of postoperative infection and wound oozing; moreover, no tendon rupture was reported in the included studies. All these results suggest that the drug composition and amount of steroids in the included studies did not increase the risk of postoperative infection and wound problems. Although existing studies have not demonstrated a significant increase in the incidence of such catastrophic complications, MCPI with steroids should be best used in patients undergoing primary TKA for osteoarthritis; patients with a history of aforementioned ailments should be excluded [11].

In spite of previous studies which presumed that MCPI with steroids might decrease the blood loss due to a reduction in the production of prostaglandins with vasodilatory effects [11, 12, 15], the result of our meta-analysis showed that it could not significantly reduce the postoperative drainage. One thing to note here is that postoperative drainage only represents obvious blood loss; whether the total blood loss is reduced due to MCPI with steroids is still unknown. For this reason, RCTs which evaluate the changes of hemoglobin level should be performed as the existing data could not assess the changes of hidden and total blood loss by meta-analysis.

The limitations of our study include the following. (1) The meta-analysis of ROM and some postoperative VAS appeared heterogeneous, and sensitivity analysis and subgroup analyses failed to eliminate the heterogeneity. As those results of meta-analysis had clinical agreement, we included all those studies and conducted the meta-analysis by the random-effects model for the reason that those studies were of high quality, which may slightly influence the reliability of the meta-analysis. (2) The included studies were lacking in, inflammation evaluating indexes like interleukin-6, serum C-reactive protein, and erythrocyte sedimentation rate, so we could not evaluate the efficacy of MCPI with steriod comprehensively.

## Conclusions

For patients undergoing TKA, the addition of steroids, which is proved to be highly safe, further improves the analgesic efficacy of MCPI. MCPI with steroids might neither increase the early or long-term postoperative ROM of knee nor may it reduce the postoperative drainage. However, the duration of time required to perform straight-leg raising and length of hospital stay are reduced. However, the best results are acquired in patients without any altered immunological status. New RCTs which evaluate the changes in hematocrit levels and those which incorporate knee-score system should be performed for the purpose of evaluating the efficacy of MCPI with steriod comprehensively.

## Abbreviations

TKA: total knee arthroplasty
MCPI: multimodal cocktail periarticular injection
ROM: range of Motion
RCTs: randomized controlled trials
VAS: visual analogue scale
SLR: straight-leg raise
RR: relative risk
WMD: weighted mean difference
CI: confidence interval
